# Implementation of a Real-Time Object Pick-and-Place System Based on a Changing Strategy for Rapidly-Exploring Random Tree

**DOI:** 10.3390/s23104814

**Published:** 2023-05-16

**Authors:** Ching-Chang Wong, Chong-Jia Chen, Kai-Yi Wong, Hsuan-Ming Feng

**Affiliations:** 1 Department of Electrical and Computer Engineering, Tamkang University, New Taipei City 25137, Taiwan; wong@ee.tku.edu.tw (C.-C.W.);; 2 Department of Electrical Engineering, Chung Yuan Christian University, Taoyuan City 32023, Taiwan; 3 Department of Computer Science and Information Engineering, National Quemoy University, Kinmen County 892, Taiwan; hmfeng@nqu.edu.tw

**Keywords:** rapidly-exploring random tree (RRT), path planning, robot manipulator, object pick-and-place, collision-free, robot operating system (ROS)

## Abstract

An object pick-and-place system with a camera, a six-degree-of-freedom (DOF) robot manipulator, and a two-finger gripper is implemented based on the robot operating system (ROS) in this paper. A collision-free path planning method is one of the most fundamental problems that has to be solved before the robot manipulator can autonomously pick-and-place objects in complex environments. In the implementation of the real-time pick-and-place system, the success rate and computing time of path planning by a six-DOF robot manipulator are two essential key factors. Therefore, an improved rapidly-exploring random tree (RRT) algorithm, named changing strategy RRT (CS-RRT), is proposed. Based on the method of gradually changing the sampling area based on RRT (CSA-RRT), two mechanisms are used in the proposed CS-RRT to improve the success rate and computing time. The proposed CS-RRT algorithm adopts a sampling-radius limitation mechanism, which enables the random tree to approach the goal area more efficiently each time the environment is explored. It can avoid spending a lot of time looking for valid points when it is close to the goal point, thus reducing the computing time of the improved RRT algorithm. In addition, the CS-RRT algorithm adopts a node counting mechanism, which enables the algorithm to switch to an appropriate sampling method in complex environments. It can avoid the search path being trapped in some constrained areas due to excessive exploration in the direction of the goal point, thus improving the adaptability of the proposed algorithm to various environments and increasing the success rate. Finally, an environment with four object pick-and-place tasks is established, and four simulation results are given to illustrate that the proposed CS-RRT-based collision-free path planning method has the best performance compared with the other two RRT algorithms. A practical experiment is also provided to verify that the robot manipulator can indeed complete the specified four object pick-and-place tasks successfully and effectively.

## 1. Introduction

Due to the presence of various objects in the working environment, path planning for robots is one of the most important topics in robotics research and is widely discussed [1,2,3,4]. If the robot does not have a good path planning method to choose a collision-free path, various collision situations may occur. Once a collision occurs, unpredictable or large losses may be caused. Therefore, many researchers devote themselves to the field of collision-free path planning. Based on differential search methods, path planning algorithms are divided into three categories: search-based, heuristic-based, and sampling-based. The A* algorithm proposed by Hart et al. is a search-based path planning algorithm [5]. It first models the environment and then determines objective node information to avoid ineffective exploration of the environment and find a better solution efficiently. However, when exploring high-dimensional spaces or wide environments, the computing time of the A* algorithm increases significantly. The ant colony system proposed by Dorigo et al. is a heuristic-based path planning algorithm [6]. It finds a better solution through a function, iteration by iteration, in each exploration of the environment. However, the convergence speed of the heuristic-based algorithm cannot be guaranteed. Due to the long computing times of search-based and heuristic-based path planning algorithms, they are not suitable for real-time system applications. The probabilistic roadmap algorithm proposed by Kavraki et al. is a sampling-based path planning algorithm [7]. The advantage of the sampling-based algorithm is that it reduces the burden of modeling the environment by using sampling points to scatter the entire space into a partial area of the environment. This makes it easy to represent all the features of the environment. It can handle the path planning of robots in high-dimensional spaces. However, the search efficiency and success rate of the probabilistic roadmap will decrease when there are dense obstacles in space. The rapidly-exploring random tree (RRT) proposed by LaValle is also a sampling-based path planning algorithm [8]. It combines the advantages of sampling and searching abilities in the environment. Random trees will randomly expand new nodes in the environment. This algorithm not only inherits the advantages of the fast search speed of the probabilistic roadmap algorithm but can also perform a wide range of exploration in the environment, which is more powerful to deal with the online path planning of high-dimensional spaces. 

Although the basic RRT algorithm can find a better path in the search space relatively quickly, it still has some problems that need to be improved, such as spending a lot of time exploring some invalid areas. Therefore, there have been many studies aimed at improving the basic RRT algorithm. For example, Wang et al. proposed an RRT algorithm based on a node control mechanism [9]. Based on this mechanism, the node expansion of the random tree is constrained by defined conditions, which reduces the generation of invalid nodes and thus finds a better solution more efficiently than most RRT algorithms, especially in narrow areas of the search space. Kang et al. proposed an RRT algorithm based on a goal-oriented mechanism [10]. It improves the search efficiency by increasing the sampling probability of the search point that is near the target. In addition, the sampling strategy is appropriately switched through the node counting mechanism to adapt to the complex environment. In addition, sometimes the RRT algorithm will overfocus on the goal area, which makes it difficult to find a path when encountering a complex environment. On the other hand, if the algorithm only focuses on improving its adaptability, it will not be fast enough to find a path to the goal in simple environments. Therefore, there are still many ways to improve the path planning for robot applications. In order to improve the computing time and environmental adaptability of the existing RRT algorithm, an improved RRT algorithm is proposed in this paper.

Path planning is important for any robot. In addition, we can find a wide range of industrial applications for robot pick-and-place operations on robot manipulators [11,12,13]. This research includes the discussion of path planning, object picking and placing, collision avoidance, and control of the robot manipulator. Many improved RRT algorithms have been used in robot manipulators to achieve good results. However, most of them only established a simulated environment to present simulation results. In order to illustrate the proposed RRT algorithm, let a real robot manipulator perform object pick-and-place tasks in real time. The robot operating system (ROS) is used to design and integrate the hardware and software of an object pick-and-place system. Since ROS can transmit or receive different types of data at the same time through “messages” and “services”, it is one of the most popular platforms for research in robotics. Moreover, MoveIt, which is open-source motion planning software, has been widely used in industry and research. It is easy to integrate with ROS to set up new robots, and it is already available for more than 150 robots. Therefore, in the design of motion planning, MoveIt is used to complete the required motion of the robot manipulator.

There are six sections in this paper. In Section 1, the background is introduced. In Section 2, an implemented object pick-and-place system based on ROS is described. In Section 3, three sampling strategies used in the RRT algorithms are described. In Section 4, a basic RRT algorithm and an improved RRT algorithm based on an existing RRT algorithm are described. In Section 5, an experimental environment with four pick-and-place tasks is setup, and some simulation and actual experimental results are presented to illustrate that the implemented object pick-and-place system using the proposed RRT algorithm can allow a robot manipulator to pick and place objects in real time. Finally, conclusions and future work are described in Section 6.

## 2. ROS-Based Object Pick-and-Place System

**Figure 1:**
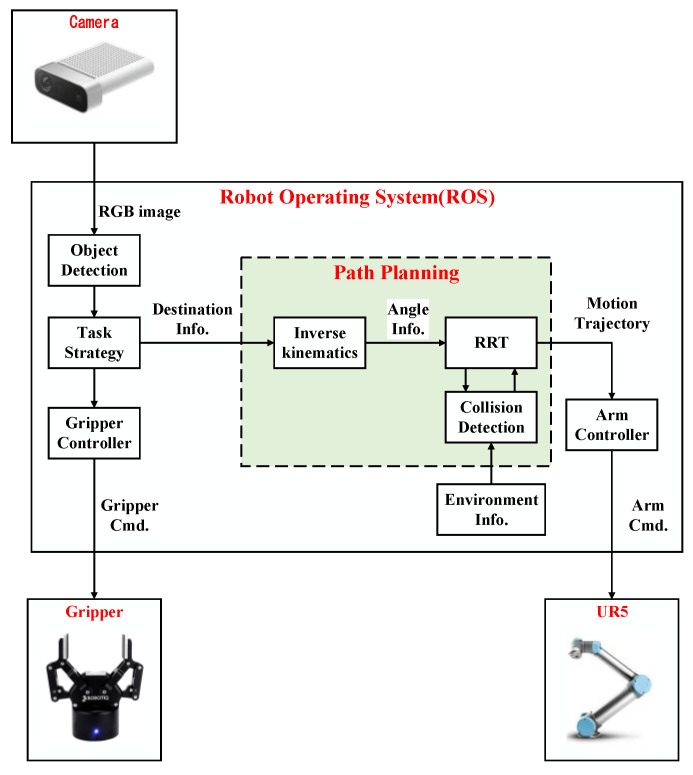
System diagram of the proposed ROS-based object pick-and-place system.

The system architecture diagram of the implemented ROS-based object pick-and-place system is shown in Figure 1. The input of this system is the RGB image captured by Microsoft Azure Kinect DK (a RGB-D camera), and the outputs are the control commands of Universal Robots UR5 (six-degree-of-freedom (DOF) robot manipulator) and Robotiq 2F-85 (a two-finger parallel gripper). Azure Kinect DK has the features of a wide field of view and easy installation; it is directly installed on a bracket to capture images on the table. UR5 has some features, such as a light weight, a user-friendly interface, and collision detection capability. The related unified robot description format (URDF) files for the specific MoveIt applications are also provided. These features make experiments easy to perform and avoid collisions between the robot manipulator and surrounding objects during the experiment. Robotiq 2F-85 is easily integrated into robot manipulators. It has the feature that it can avoid damage to the gripper itself and prevent the robot manipulator from injuring the object during the grasping task.

The object detection module is implemented by the you only look once (YOLO) algorithm [14] to obtain the position information of the object on the table from the captured RGB image. The task strategy module is used to decide the destination of the robot manipulator when a pick-and-place task is given. The path planning module is implemented by the improved RRT algorithm, which can quickly select a collision-free path so that the robot manipulator and the two-finger gripper can successfully complete the given tasks in real-time.

In the system integration of the proposed pick-and-place system, ROS is used to handle the communication between each module. The robot motion information is calculated by MoveIt through ROS. The version of ROS Melodic with Ubuntu 18.04 is used. MoveIt is an open-source motion planning software that is a state-of-the-art implementation of robot motion and path planning. Thus, it is used for the motion planning of the robot manipulator. MoveIt provides a variety of functional packages for users to choose from and integrates various functional plugins such as kinematics, collision detection, and motion planning so that it can provide the desired motion planning for various robots. Moreover, MoveIt is a package of ROS, and it is highly integrated with ROS so that the results of motion planning by MoveIt can be easily transmitted to the robot manipulator through ROS. Users can use the 3D visualization tool RViz to visually present the motion planning results in the ROS. For MoveIt, the unified robot description format (URDF) and semantic robot description format (SRDF) are used to describe robots. The proposed RRT algorithm is designed for the robot manipulator UR5 in this paper, but it can be used for the other six DOF robot manipulators. MoveIt imports the URDF file to set the parameters of the robot and the simulated environment, then sends a request to the default library, the open motion planning library (OMPL), to design a suitable motion trajectory. After calculating a path, MoveIt will divide this path into the same distances and add information such as speed, acceleration, and the consumed time of the robot at each piece of the path. In addition, OMPL is the main library of sampling-based planning algorithms, which includes many modules of common RRT algorithms. Because of its modular program design, it is easy for users to add custom motion planning algorithms. Therefore, MoveIt is adopted as the motion planning software for the robot manipulator in this paper.

As shown in Figure 1, the image information is sent by the object detection module, and the motion information is calculated by MoveIt through ROS. Since ROS can transmit or receive different data through messages and services, the proposed system integrated through ROS can be applied to various input and output devices, so it has good applicability. In the communication mode between the object detection module, task strategy module, MoveIt, and UR5, there are mainly three two-way communication services in the implemented pick-and-place system, which are, respectively, named Service1, Service2, and Service3. 

In order to ensure that the task strategy module can indeed receive the object position information from the object detection module, Service1 is used to make the control command of the task strategy module for the robot manipulator to move only after it has received the object position information. The nodes of the server and client of Service1 are the task strategy module and the object detection module, respectively. The request sent by the task strategy module to the server has a status value of 0 (false) or 1 (true) while the control command is received. The response is given by the object detection module as the client after it receives the request for the object coordinates (x, y) on the table. The nodes of the server and client of Service2 are the task strategy module and MoveIt, respectively. The request sent by the task strategy module as the server is the target position (x, y, z) of the end effector and the quaternion of the robot manipulator pose (w, x, y, z). The response given by MoveIt as the client after receiving the request is the result of forward and inverse kinematics and the motion trajectory obtained by RRT. The nodes of the server and client of Service3 are MoveIt and UR5, respectively. The request sent by MoveIt as the server is the joint motion trajectory of the robot manipulator. The response given by UR5 as the client after receiving the request is a status value of 0 (false) or 1 (true), depending on whether UR5 is busy or not.

## 3. Sampling Strategy

**Figure 2:**
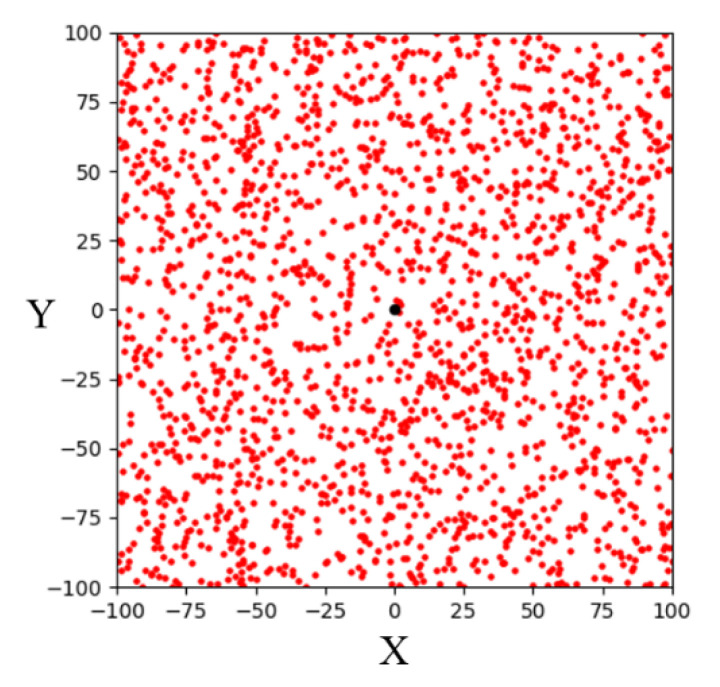
Schematic illustration of random sampling in a two-dimensional space.

Random sampling is the sampling strategy used by the basic RRT algorithm. The algorithm randomly samples the entire space to determine sampling points. The simulation result of 1000 random samplings in a 200 × 200 two-dimensional space is shown in Figure 2, where the black point is the goal point. It can be seen that the sampling points randomly fall throughout the entire space. For the RRT algorithms, random sampling can fully explore the environment, find a path from the start point to the goal point, and avoid obstacles in the environment. However, due to its randomness, it spends a lot of time exploring invalid areas in most environments. Therefore, many improved sampling strategies were proposed to reduce computing time.

**Figure 3:**
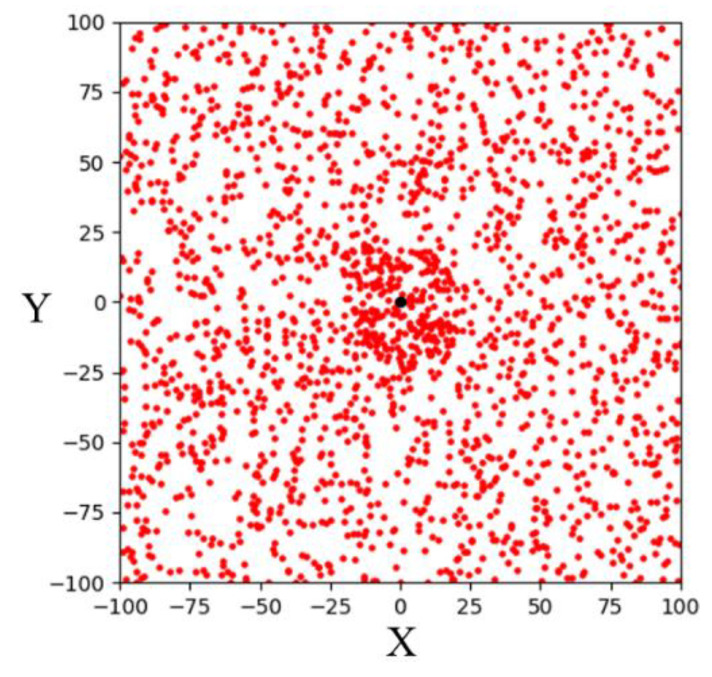
Schematic illustration of goal-biased sampling in a two-dimensional space.

Goal-biased sampling is an improved sampling strategy over random sampling. By adding a random variable rand and set: if rand is less than the specified probability p, then the point will be selected as a sampling point. Otherwise, use the original random sampling method to randomly sample the space. This sampling strategy causes the random tree of the RRT algorithm to take the goal point to sample with a certain probability. In this case, random trees can approach the goal area faster and reduce computing time while maintaining random sampling to fully explore the environment. The simulation result of 1000 goal-biased sampling points in a 200 × 200 two-dimensional space is shown in Figure 3. It can be seen that more sampling points fall within the goal area.

**Figure 4:**
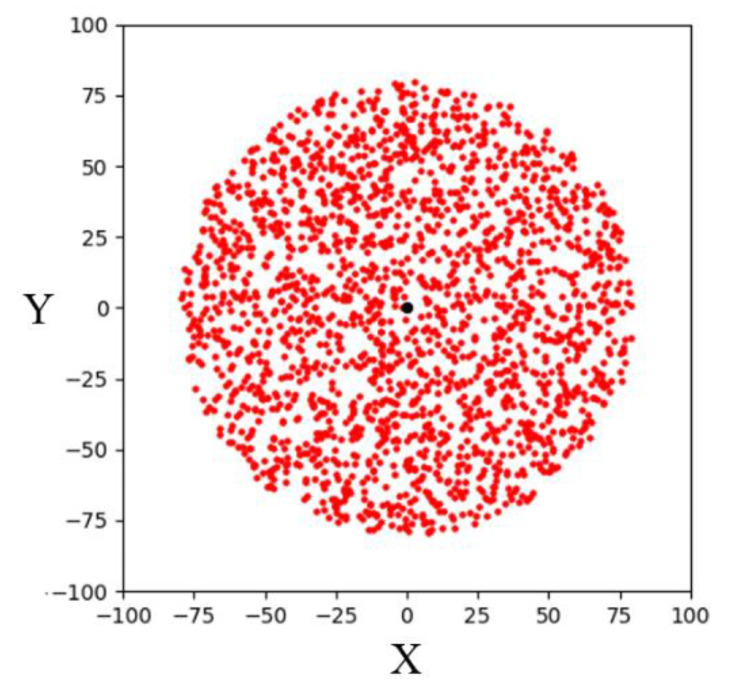
Schematic illustration of bounded sampling in a two-dimensional space.

Bounded sampling is a sampling strategy to limit the sampling area to a radius from the goal point and let the random tree explore toward the goal area. The simulation result of 1000 bounded sampling in a 200 × 200 two-dimensional space is shown in Figure 4. It can be seen that the sampling points are limited within a radial space centered on the goal point. Compared with the RRT algorithm using a goal-biased sampling strategy, the RRT algorithm using bounded sampling can make the random tree explore the goal area stably. By gradually reducing the sampling radius, this algorithm can find a path to the goal point. However, since such bounded sampling may be overly focused on exploring towards the goal area, random trees can easily get trapped in some complex environments. 

## 4. Changing Strategy RRT Algorithm

In the design of the sampling-based path planning method, two items, such as computing time and path length, are usually considered. For offline path planning, the path length is usually the main consideration. On the other hand, for online path planning, computing time is the priority consideration. Real-time object picking and placing tasks require online path planning, so we mainly focus on how to reduce the computing time of the improved RRT algorithm.

**Figure 5:**
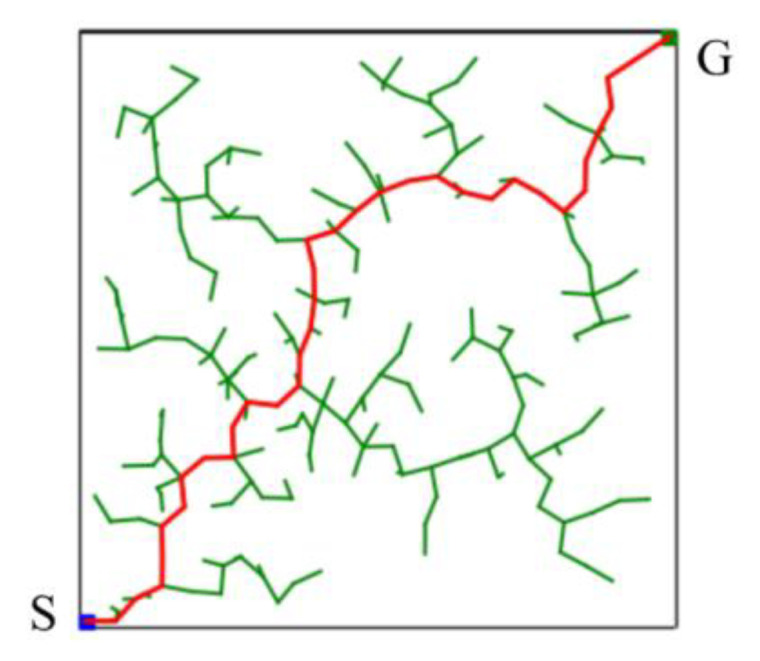
Schematic illustration of the basic RRT algorithm for finding a solution in a two-dimensional space.

The basic RRT algorithm, as a sampling-based path planning method, is mainly designed to perform random sampling in the configuration space [15]. A schematic illustration of the basic RRT algorithm for finding a path from a starting point (S) to a goal point (G) in a two-dimensional space is shown in Figure 5. It can be seen that the random tree fully explores the environment. The advantage of this method is that it does not require modeling the entire environment. Such path planning algorithms can explore two-dimensional spaces faster than other path planning algorithms. Therefore, it is suitable for solving the path planning problem in complex or constrained environments. 

The proposed algorithm is named changing strategy RRT (CS-RRT), which is improved on the basis of the method of gradually changing the sampling area based on RRT (CSA-RRT) [9]. The pseudocode of the CSA-RRT algorithm is shown in Algorithm 1. It needs to calculate the distance $D_{max}$ between the two nodes $q_{far}$ and $q_{goal}$, where $q_{far}$ is the node farthest from the goal point $q_{goal}$. Since the tree only has the initial node $q_{start}$ at the beginning, the algorithm will initially use $q_{start}$ as $q_{far}$ to calculate $D_{max}$, which is the initial sampling radius R of bounded sampling. When the dimension of the space is s, the maximum distance $D_{max}$ is calculated by:

(1) $$D_{max}=\sqrt{{{(q}_{goal(1)}-q_{far(1)})}^{2}+\ldots +{{(q}_{goal(s)}-q_{far(s)})}^{2}}$$

The CSA-RRT algorithm uses the random sampling method to randomly select a sampling point $q_{rand}$ in the space. The distance $D_{rand}$ between $q_{rand}$ and $q_{goal}$ is calculated by:

(2) $$D_{rand}=\sqrt{{{(q}_{goal(1)}-q_{rand(1)})}^{2}+\ldots +{{(q}_{goal(s)}-q_{rand(s)})}^{2}}$$

**Figure 6:**
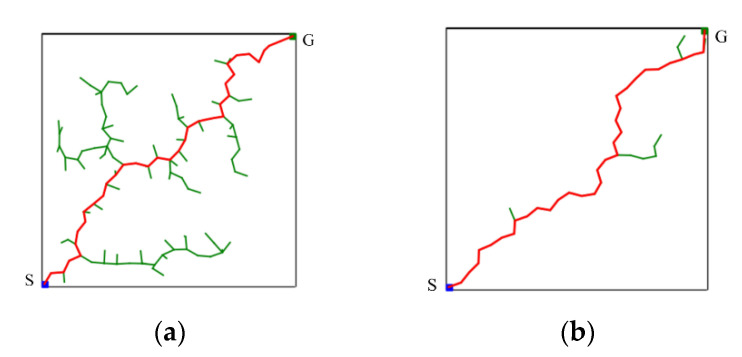
Schematic illustration of the comparison results of the two RRT algorithms. (**a**) 10% goal-biased RRT algorithm. (**b**) CSA-RRT algorithm.

Compare the distance between $D_{rand}$ and R to make sure that the sampling point $q_{rand}$ is inside R. If $D_{rand}$ is less than R, then $q_{rand}$ is considered a valid sampling point. Conversely, if $D_{rand}$ is greater than R, it means that $q_{rand}$ is outside R, and the algorithm will resample until $q_{rand}$ is inside R. If a new node $q_{new}$ is successfully added to the random tree in an iteration, it means that there is no obstacle between $q_{new}$ and the nearest node $q_{near}$. When the new point $q_{new}$ is closer to the goal point $q_{goal}$, it becomes the nearest node. At this time, the value of R is changed to the distance from the new nearest node $q_{new}$ to the goal point $q_{goal}$. Conversely, it means that an obstacle is encountered during the expansion process. At this time, a step size ε of k times is added to R, which means that the sampling area is expanded so that the random tree can avoid nearby obstacles. The value of k is a positive integer for adjusting the sampling radius, which needs to be manually adjusted according to the complexity of the environment. This allows the algorithm to explore the direction of $q_{goal}$ as much as possible while having the ability to randomly explore the environment. The comparison results of the CSA-RRT algorithm and the 10% goal-biased RRT algorithm are shown in Figure 6. It can be seen that the CSA-RRT algorithm can reduce the generation of invalid nodes more than the goal-biased RRT algorithm.

**Algorithm 1:** CSA-RRT algorithm

T ← InitTree($q_{start}$);
R ← $D_{max}$;
**for** i = 1 **to** n **do**

 $q_{rand}$ ← RandomSample();

 **if** Distance($q_{rand}$,$q_{goal}$) > R **then**

  **continue**;

 **end if**

 $q_{near}$ ← NearestNeighbor($q_{rand}$, T);

 $q_{new}$ ← Extend($q_{rand}$,$q_{near}$, ε);

 **if** CollisionFree($q_{near}$,$q_{new}$) **then**

  AddNewNode(T,$q_{new}$);

  R ← Distance($q_{new}$,$q_{goal}$);

 **else**

  R ← R + k × ε;

  **continue**;

 **end if**

 **if** Distance($q_{new}$,$q_{goal}$) < $\rho_{min}$ **then**

  **return** T;

 **end if**

**Figure 7:**
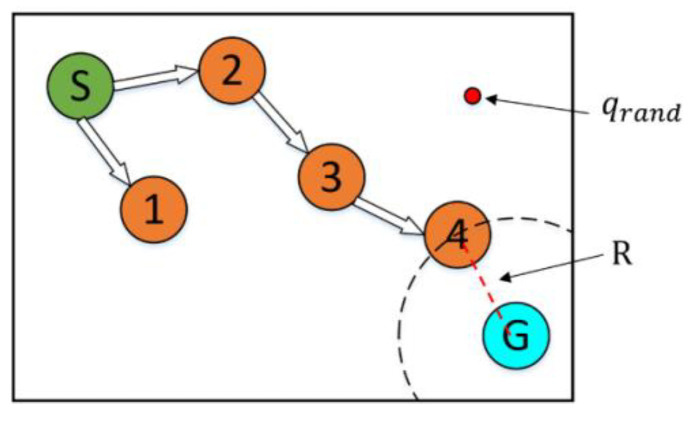
Schematic illustration of the sampling point. It is difficult to fall within the sampling radius *R* when the new point is close to the goal point.

**end for**
**return** Failed;



The CSA-RRT algorithm has the advantage that the invalid nodes of the CSA-RRT algorithm are much lower than those of the goal-biased RRT algorithm. However, the computing time of the CSA-RRT algorithm is not much faster than that of the goal-biased RRT algorithm. After observation, we found that although the sampling radius R will gradually shrink as $q_{new}$ gets closer to the goal area, thereby reducing the generation of invalid points. However, on the other hand, because of the reduction in R, $q_{rand}$ selected by random sampling becomes more and more difficult to fall within R. As shown in Figure 7, when the new point $q_{new}$ is close to the goal point $q_{goal}$, R will become smaller and smaller. This results in a very small chance that the sampling point $q_{rand}$ will fall within R. As a result, the CSA-RRT algorithm spends a lot of time doing computation at certain stages. Therefore, the CSA-RRT algorithm has the advantage of generating fewer invalid nodes, but it still cannot significantly reduce the computing time of path planning. This becomes more apparent when sampling in larger environments. 

A sampling-radius limitation mechanism is adopted to solve this problem that $q_{rand}$ is difficult to fall into R when $q_{new}$ is close to $q_{goal}$. An additional statement is used to determine whether the random tree is approaching $q_{goal}$. Whenever $q_{new}$ is added to the random tree, *R* and $D_{max}$ are compared before the next sampling. If R is greater than one-fifth of $D_{max}$, it means that the random tree is still far away from $q_{goal}$. Thus, it continues to use the random sampling of the CSA-RRT algorithm to select $q_{rand}$. On the other hand, if *R* is smaller than one-fifth of $D_{max}$, it means that the random tree is close to $q_{goal}$. At this time, a sampling-radius limitation mechanism based on bounded sampling is adopted to limit the sampling area within the radius R from the goal point. In this way, the problem that $q_{rand}$ cannot successfully fall within R when it is close to the goal point can be solved. This makes the proposed CS-RRT algorithm not only quickly find valid nodes but also reduce the computing time of path planning. In the case of two-dimensional simulations, the CS-RRT algorithm improves by about 0.5 times compared with the CSA-RRT algorithm.

**Figure 8:**
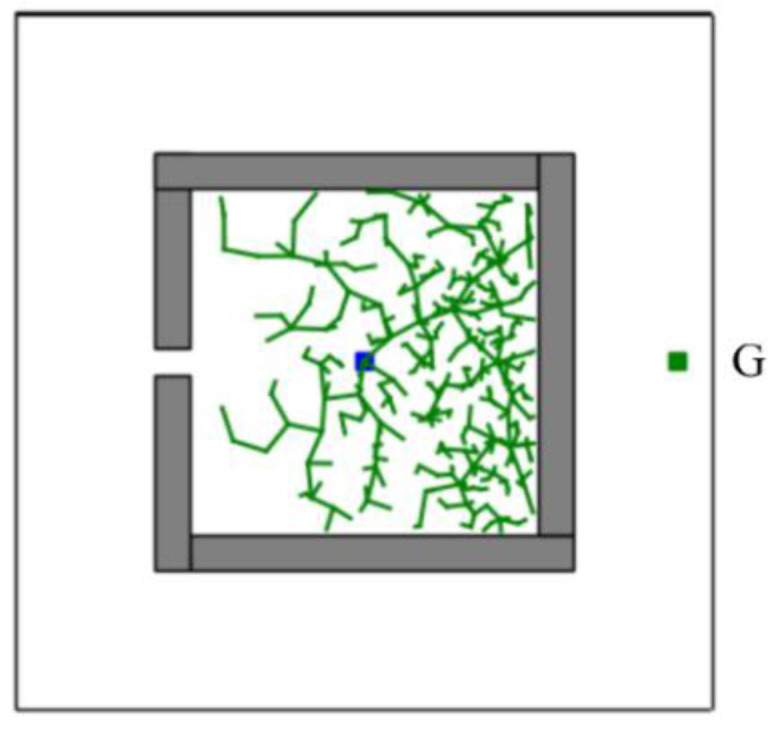
Schematic illustration of CSA-RRT algorithm trapped in a complex environment with a restricted region.

In addition, the CSA-RRT algorithm has the advantage that it can quickly find an initial path to the goal area. However, as shown in Figure 8, if the CSA-RRT algorithm is performed in a complex environment and the value of k is not adjusted properly, the tree may be trapped due to focusing too much on the goal point. As a result, the random tree keeps expanding in the same area but cannot find an escape path until the number of node expansions of the algorithm reaches the maximum limit of expansions and fails. In order to solve this problem, a node counting mechanism is adopted to appropriately switch the sampling strategy to an appropriate sampling method in complex environments. It can avoid the search path being trapped in some constrained areas and improve the adaptability of the proposed CS-RRT algorithm to various environments.

The CSA-RRT algorithm with the node counting mechanism will calculate the distance $D_{min}$ from the node $q_{min}$ that is closest to the goal point $q_{goal}$ and set the node count variable nodecnt to zero during initialization. As shown in Algorithm 2, in the sampling stage, the algorithm will choose which sampling method to use according to the value of nodecnt. If nodecnt is less than the set threshold, the sampling method of CSA-RRT is used to make the random tree quickly extend to the goal point. Otherwise, the random sampling method, which fully explores the environment, is used.

**Algorithm 2:** SelectSample(
nodecnt
**,**
$q_{goal}$
**,**
R
);

**if** nodecnt < 20 **then**

 $q_{rand}$ ← RandomSample();

 **if** Distance($q_{rand}$,$q_{goal}$) > R **then**

  **continue**;

 **end if**

**else**

**Figure 9:**
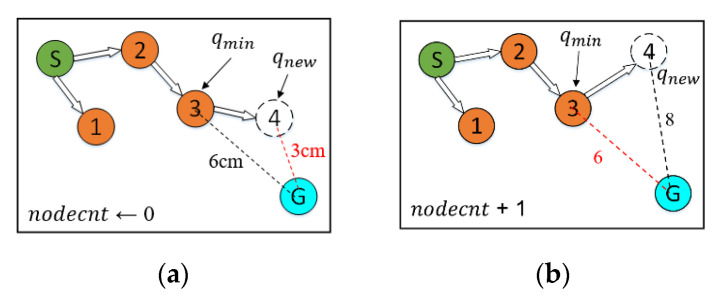
Schematic illustration of two different node number adjustments. (**a**) The new node is close to the goal point. (**b**) The new node is far from the goal point.

 $q_{rand}$ ← RandomSample();

**end if**
**return** $q_{rand}$;



After completing the collision detection stage in each iteration, the algorithm calculates the Euclidean distance $D_{new}$ from $q_{new}$ to $q_{goal}$, no matter if $q_{new}$ is successfully added to the random tree. After this, compare $D_{new}$ with $D_{min}$ in the CheckEnvironment() function. The pseudocode of the CheckEnvironment() function is shown in Algorithm 3. If $D_{new}$ is smaller than $D_{min}$, then nodecnt is set to zero. At this time, $q_{new}$ is closer to $q_{goal}$, which means that the tree is approaching the goal area, so there is no need to change the sampling strategy. After that, change $q_{new}$ into $q_{min}$ as the basis for the next check of the expansion status. On the other hand, if $D_{new}$ is greater than $D_{min}$, it means that $q_{new}$ is not closer to $q_{goal}$. At this time, nodecnt+1. If $D_{new}$ continues to be greater than $D_{min}$ for the next few times, it is considered that the random tree is trapped in the current area. Then the algorithm will switch the sampling method to random sampling in the SelectSample() function to try to escape the current area until $D_{new}$ is smaller than $D_{min}$. In addition, an upper limit is set to avoid the algorithm wasting too much time using random sampling to explore the space. Therefore, when nodecnt reaches the set upper limit, it will reset to zero immediately. A schematic illustration of node count adjustment is shown in Figure 9.

**Algorithm 3:** CheckEnvironment(
$D_{new}$, $D_{min}$, nodecnt
)

**if** $D_{new}$ < $D_{min}$ **then**

 $D_{min}$ ← $D_{new}$;

 nodecnt ← 0;

**else**

 nodecnt ← nodecnt + 1;

**end if**
**if** nodecnt > 100 **then**

 nodecnt ← 0

**end if**



**Figure 10:**
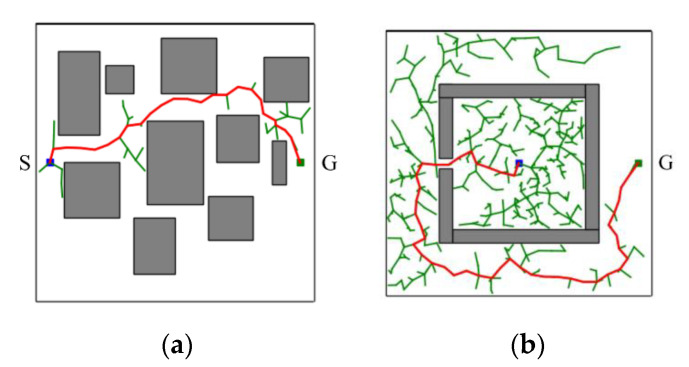
Schematic illustration of the performance of CS-RRT algorithm in two different environments. (**a**) A simple environment with many obstacles. (**b**) A complex environment with a restricted region.

In short, the proposed CS-RRT algorithm is based on the CSA-RRT algorithm and uses the sampling-radius limitation mechanism and the node counting mechanism to solve the problems existing in the CSA-RRT algorithm. The sampling-radius limitation mechanism allows the random tree to finish the sampling stage more quickly when it is close to the goal area, so that the proposed CS-RRT algorithm can further reduce the computing time. The node counting mechanism makes the algorithm avoid overfocusing on the goal area, so the proposed CS-RRT algorithm also has good adaptability to the environment. The results of the proposed CS-RRT algorithm performed in two different environments are shown in Figure 10. When encountering simple environments, as shown in Figure 10a, the proposed CS-RRT algorithm can quickly find a path. When encountering complex environments, as shown in Figure 10b, the proposed CS-RRT algorithm can also prevent trapping by switching sampling strategies. Comparing the results shown in Figure 8, we can see that the CSA-RRT algorithm is trapped in this environment. With these improvements, the proposed CS-RRT algorithm indeed not only reduces computing time but also improves environmental adaptability.

## 5. Simulation Results and Experimental Results

### 5.1. Simulation Results of Robot Manipulator

**Figure 11:**
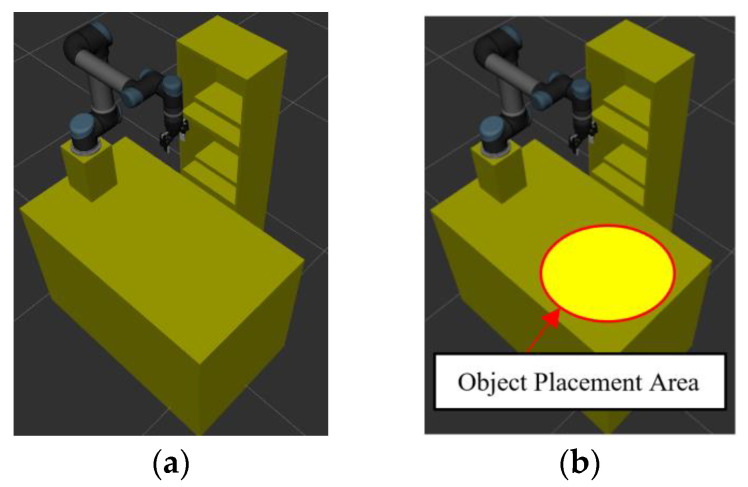
Schematic illustration of experimental environment. (**a**) The robot manipulator and experimental environment. (**b**) An object will be randomly placed inside the red circle.

The simulation setup of the robot manipulator and experimental environment is shown in Figure 11. In order to demonstrate the performance of the motion planning of the robot manipulator based on the proposed CS-RRT algorithm, a series of tasks (Task 1~Task 4) is designed, and then the success rates and average computing time after 50 experiments of each algorithm, the proposed CS-RRT, CSA-RRT, and the 10% goal-bias RRT algorithm, provided by the open motion planning library (OMPL), are compared. The tasks are described as follows:

Task 1: The implemented pick-and-place system uses the YOLOv4 algorithm to obtain the position of Object A on the table and then commands the robot manipulator to grasp the Object A. This task verifies the motion planning performance of algorithms in an open space without obstacles.
Task 2: The implemented pick-and-place system commands the robot manipulator to place the grasped object at a certain location on the upper layer of the cabinet. The sides of the cabinet can be considered obstacles for the movement of the robot manipulator. This task verifies the motion planning performance of algorithms from open space to restricted space.
Task 3: The implemented pick-and-place system commands the robot manipulator to move from the upper layer of the cabinet to the lower layer and grasp the Object B that was placed on the lower layer of the cabinet. This task verifies the motion planning performance of algorithms in two restricted spaces.
Task 4: The implemented pick-and-place system commands the robot manipulator to move from the lower layer of the cabinet to the grasping position in Task 1 and to place the grasped object. This task verifies the motion planning performance of algorithms from a restricted space to an open space.

**Figure 12:**
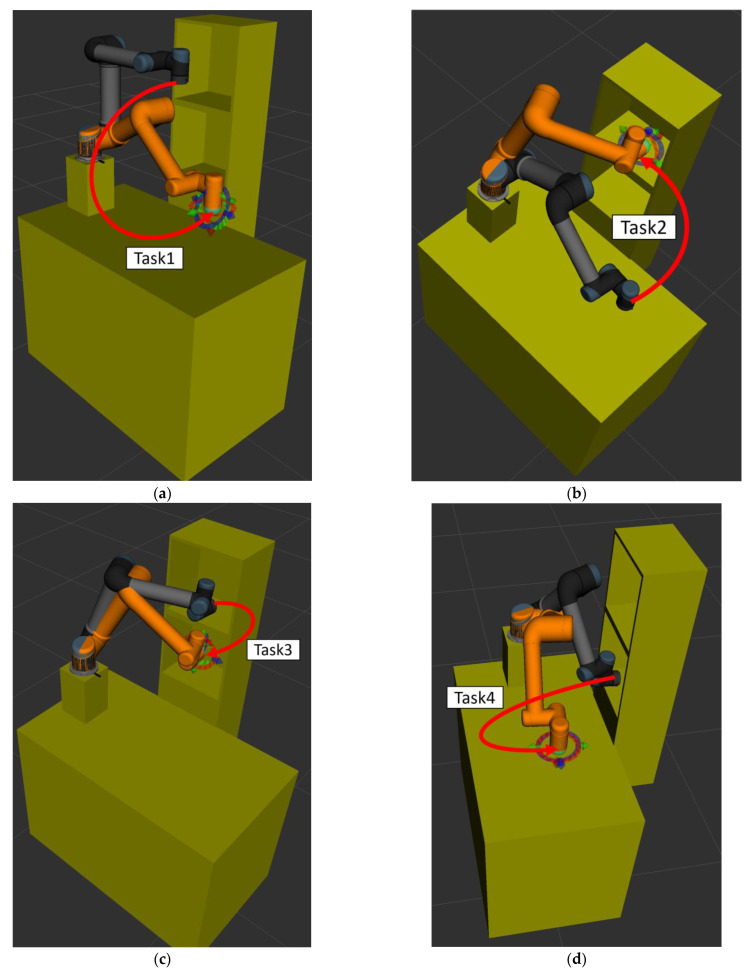
Schematic illustration of the experimental scenarios for four tasks. (**a**) The motion flow of the manipulator in Task 1. (**b**) The motion flow of the manipulator in Task 2. (**c**) The motion flow of the manipulator in Task 3. (**d**) The motion flow of the manipulator in Task 4.

**Figure 13:**
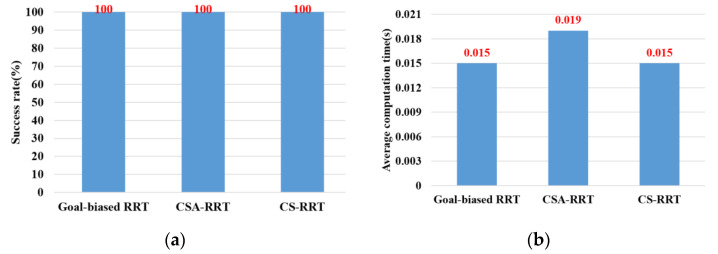
Experimental results of three algorithms in the scenarios of Task 1. (**a**) Success rate. (**b**) Average computing time.

Figure 12 illustrates the motion flow from Task 1 to Task 4. Note that the gray and orange robot manipulators indicate the initial and finish positions of each task, respectively. Four simulation results for each task are illustrated as follows:

In the experimental scenarios of Task 1, as shown in Figure 12a, the robot manipulator is commanded to move from its initial position to grasp the object on the table. Figure 13a,b are bar graphs of the success rate and the average computing time of the three algorithms executed 50 times in Task 1, respectively. From the results in Figure 13, it can be seen that the success rate and the average computing time of the proposed CS-RRT and the 10% goal-biased RRT are the same. We infer that the proposed CS-RRT needs to compute the sampling radius at each iteration. Therefore, searching in an open space without obstacles, does not have the advantage of taking less computing time. In addition, CSA-RRT makes it difficult for the sampling points to fall within the sampling radius when they are close to the goal area. Therefore, the computing time of the CSA-RRT is higher than that of the proposed CS-RRT.

Remark: The experiment sets the maximum path planning time to 1 second because it is basically not regarded as real-time motion planning after more than 1 second. In addition, the robot manipulator may rotate a full circle and cause damage to surrounding objects due to the randomness of the RRT algorithms. Thus, we restrict each axis of the robot manipulator to rotating between plus and minus 180 degrees.

**Figure 14:**
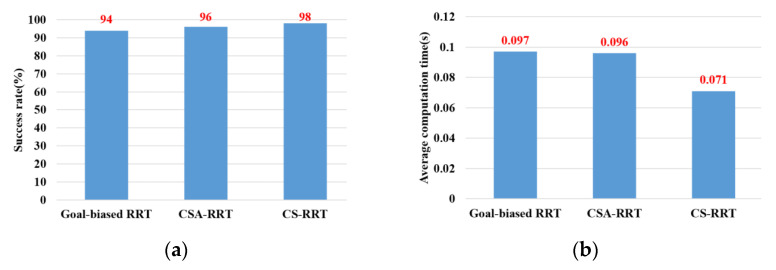
Experimental results of three algorithms in the scenarios of Task 2. (**a**) Success rate. (**b**) Average computing time.

In the experimental scenarios of Task 2, as shown in Figure 12b, the robot manipulator is commanded to move from the finished position of Task 1 to a specific position on the upper layer of the cabinet to place the grasped object. Although moving in a straight line is the fastest way to reach the destination, it will collide with the sides of the cabinet. Therefore, for the robot manipulator to reach its destination safely, it needs a collision-free path to avoid collisions with the sides of the cabinet. Task 2 tests the motion planning performance of algorithms from an open space to a restricted space. From the results shown in Figure 14, it can be seen that the proposed CS-RRT has the highest success rate and the shortest average computing time. Therefore, the proposed CS-RRT has better performance than the other two algorithms when planning a motion in a restricted environment.

**Figure 15:**
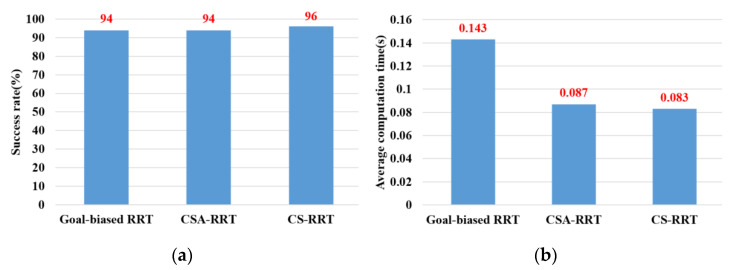
Experimental results of three algorithms in the scenarios of Task 3. (**a**) Success rate. (**b**) Average computing time.

In the experimental scenarios of Task 3, as shown in Figure 12c, the robot manipulator is commanded to move from the upper layer of the cabinet to a specific position on the lower layer of the cabinet. Since the robot manipulator has entered the upper cabinet, the environment of the two restricted areas in Task 3 is more complex than that in Task 1 and Task 2. In this experiment, we found that the default step size ε using OMPL is too small. As a result, the random tree of the three algorithms grows slowly, and the motion planning cannot be completed within the specified time. Therefore, the step size ε is increased by three times in Task 3. In other words, the expansion distance of each iteration of the random tree is increased. From the results shown in Figure 15, we can see that the proposed CS-RRT has the highest success rate and the shortest average computing time too. Therefore, the proposed CS-RRT has better performance than the 10% goal-biased RRT algorithm and CSA-RRT when planning in a complex environment.

**Figure 16:**
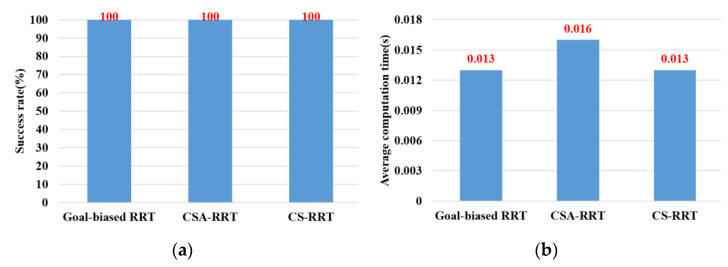
Experimental results of three algorithms in the scenarios of Task 4. (**a**) Success rate. (**b**) Average computing time.

In the experimental scenarios of Task 4, as shown in Figure 12d, the robot manipulator is commanded to move from the lower layer of the cabinet to the top of the table. This task verifies the motion planning performance of algorithms from a restricted space to an open space. From the results shown in Figure 16, we can see that the success rate and the average computing time of the proposed CS-RRT and 10% goal-biased RRT are the same. However, the average computing time of CSA-RRT under Task 4 is still longer than that of CS-RRT and 10% goal-biased RRT. Judging from the fact that the success rate of all three algorithms is 100 percent, we infer that once the robot manipulator comes out of the cabinet, there are multiple ways to move to the destination. Since the restricted area is near the initial point in Task 4, once the robot manipulator leaves the restricted area, all three algorithms can easily find a collision-free path to the destination in an open space with only a few explorations. 

Based on these results shown in Figure 13, Figure 14, Figure 15 and Figure 16, the simulation results of three algorithms in the four scenarios of Tasks 1~4 are summarized in Table 1. Compared with goal-biased RRT and CSA-RRT, the implemented pick-and-place system based on the proposed CS-RRT algorithm has a higher success rate and requires less computing time. We can see that the results of CS-RRT are similar to those of the 10% goal-biased RRT when planning is performed in non-complex environments, such as Task 1 and Task 4. However, the advantages of the proposed CS-RRT in terms of success rate and computing time can be seen when planning is performed in complex environments with space restrictions, such as Task 2 and Task 3.

### 5.2. Experimental Results of Real Robot Manipulator

**Figure 17:**
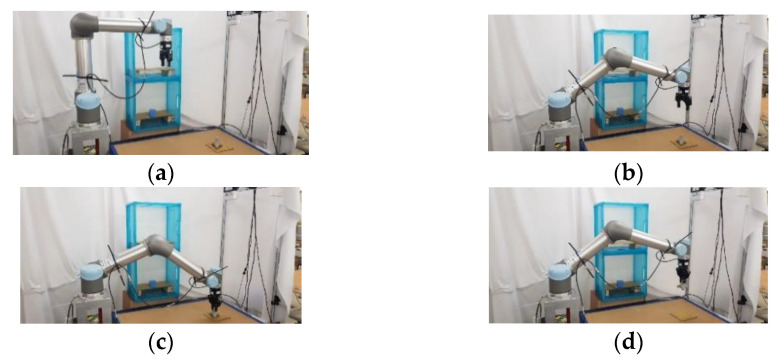
Snapshot of the robot manipulator during the object pick-and-place in Task 1. (**a**) Initial position. (**b**) Move to the top of Object A. (**c**) Move downward to grasp Object A. (**d**) Move upward to the top of the table.

**Figure 18:**
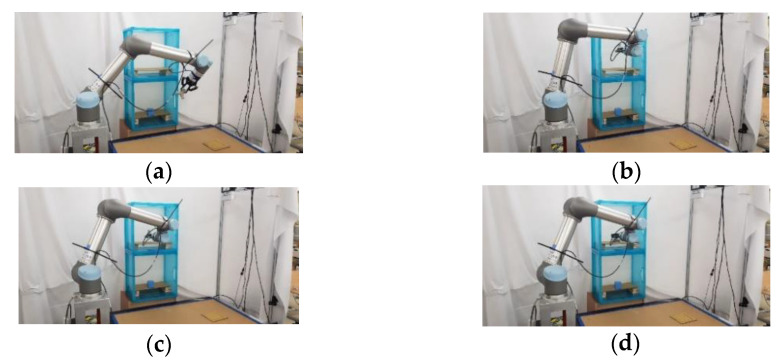
Snapshot of the robot manipulator during the object pick-and-place in Task 2. (**a**) Move to the upper layer of the cabinet. (**b**) Reach the outside of upper layer of the cabinet. (**c**) Move into the upper interior of the cabinet. (**d**) Move downward to place Object A.

**Figure 19:**
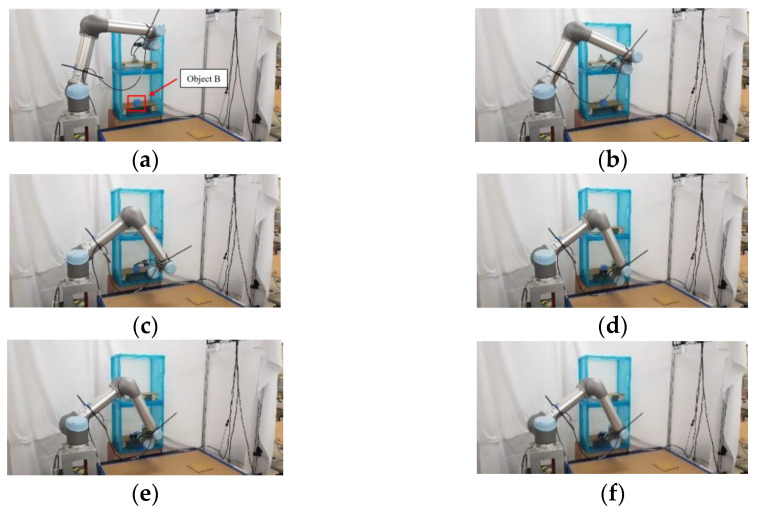
Snapshot of the robot manipulator during the object pick-and-place in Task 3. (**a**) Move back to the outside of the upper layer of the cabinet. (**b**) Move to the outside of the upper layer of the cabinet. (**c**) Move to the outside of the lower layer of the cabinet. (**d**) Reach the outside of the lower layer of the cabinet. (**e**) Move to the top of Object B, placed in the lower layer of the cabinet. (**f**) Move downward to grasp Object B.

**Figure 20:**
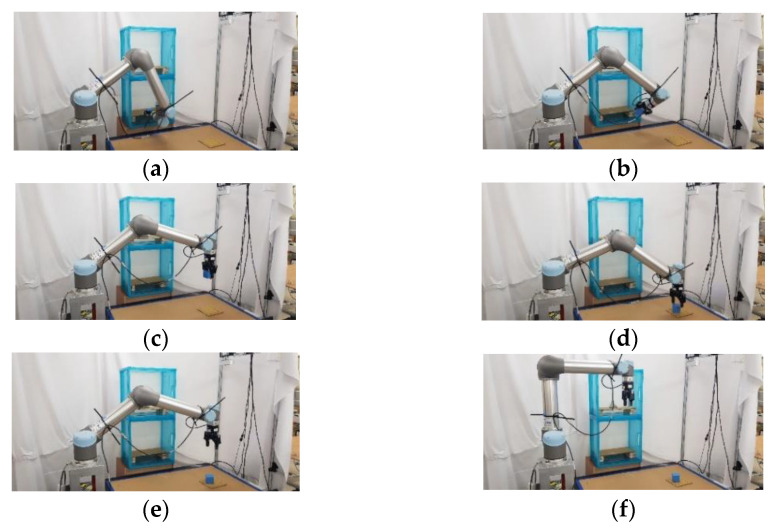
Snapshot of the robot manipulator during the object pick-and-place in Task 4. (**a**) Move back to the outside of the lower layer of the cabinet. (**b**) Move to the outside of the lower layer of the cabinet. (**c**) Reach the top of the initial position of Object A where it was originally placed on the table. (**d**) Move downward to place Object B on the table. (**e**) Move to the top of the object placed on the table. (**f**) Return to the initial position of the robot manipulator.

In the practical experimental demonstration, a real pick-and-place system with a depth camera (Microsoft Azure Kinect DK), a six DOF robot manipulator (UR5), and a two-finger parallel gripper (Robotiq 2F-85) is presented to illustrate the efficiency of the proposed CS-RRT algorithm applied in the pick-and-place system. The camera is installed above the table and the YOLOv4 algorithm is used to obtain the position coordinates of Object A, which is randomly placed on the table. Code runs on the robot with ROS implemented in Python. The communication method of ROS Services is used to ensure that the task strategy module actually receives the coordinate information and MoveIt is used to execute the motion planning results of the proposed CS-RRT algorithm to complete the four pick-and-place tasks described in the previous section. The video of the demonstration of the real pick-and-place task can be viewed on this website: https://youtu.be/lcdy2byIG_g (accessed on 20 January 2023). The snapshots of the real robot manipulator performing Task 1, Task 2, Task 3, and Task 4 are, respectively, shown in Figure 17, Figure 18, Figure 19 and Figure 20. The procedure can be described as follows:

Step 1: Obtain the position coordinates of object A randomly placed on the table through the camera installed above the table and the YOLOv4 algorithm.
Step 2: Move to the top of Object A.
Step 3: Move downward to grasp Object A.
Step 4: Move upward from the table.
Step 5: Move to the outside of the upper layer of the cabinet.
Step 6: Move into the upper interior of the upper layer of the cabinet.
Step 7: Move downward to place Object A.
Step 8: Move to the outside of the upper layer of the cabinet.
Step 9: Move to the outside of the lower layer of the cabinet.
Step 10: Move to the top of Object B in the lower layer of the cabinet.
Step 11: Move downward to grasp Object B.
Step 12: Move to the outside of the lower layer of the cabinet.
Step 13: Move to the top of the initial position of Object A, where it was originally placed on the table.
Step 14: Move downward to place Object B on the table.
Step 15: Move to the top of the object placed on the table.
Step 16: Return to the initial position of the robot manipulator.

## 6. Conclusions and Future Work

An ROS-based object pick-and-place system is implemented, and a CS-RRT algorithm is proposed so that the robot manipulator can efficiently pick-and-place objects in real time. There are three main contributions in this paper. (1) Path planning for robots is one of the most important topics in robotics research. In the research on robot manipulators for picking and placing objects in a constrained environment, most of the research only completed simulation results to verify the effectiveness of their path planning methods. Many improved RRT algorithms have been proposed, but they are rarely applied to actual robot manipulators for object pick-and-place tasks in real time. Both simulation and actual experiments are used to demonstrate that the proposed CS-RRT algorithm and the implemented system can allow the robot manipulator to effectively avoid obstacles and pick-and-place objects in real time. (2) Some disadvantages of existing RRT algorithms are addressed, and two mechanisms of sampling radius counting, and node counting are adopted in the proposed CS-RRT algorithm. The sampling-radius limitation mechanism, by limiting the sampling radius, can make the random tree finish the sampling stage faster when the tree is close to the goal point. It can reduce the computing time of the proposed CS-RRT algorithm. The node counting mechanism allows the algorithm to switch to an appropriate sampling method in a complex environment. It can avoid excessive exploration in the direction of the goal point so that the random tree does not trap itself in constrained areas. It can make the proposed CS-RRT algorithm have better environmental adaptability. In addition, an experimental environment with four object picking and placement tasks has been established. Experimental results show that the object pick-and-place system based on the proposed CS-RRT algorithm has a higher success rate and lower computing time compared with the other two path planning algorithms. (3) The robot operating system (ROS) is used to implement the object pick-and-place system. By implementing the proposed CS-RRT algorithm in the open motion planning library (OMPL), MoveIt can be used to plan the motion of the robot manipulator. According to the imported URDF file, MoveIt can also perform motion planning for different robot manipulators, so the proposed method can be easily applied to different robot manipulators.

There are two parts to the future work: (1) In the part of switching strategy and step size adjustment, switching sampling strategy can improve the adaptability of the proposed algorithm to the environment, but its own parameters need to be manually designed according to the environment. In addition, the step size also needs to be chosen according to the environment. Therefore, some optimization methods can be used in the future to select appropriate parameters for the switching strategy and step size according to the environment. (2) In the part of the sampling method where the distance needs to be calculated. In the path planning of the six-dimensional joint space of the robot manipulator, more parameters are needed to calculate the distance, which increases the computing time of the proposed algorithm. Therefore, the number of calculation distances can be reduced in the future to reduce the computing time needed to meet the system requirements.

## Figures and Tables

**Table 1:** Simulation results of three algorithms in the four scenarios of Tasks 1~4.

	Success Rate (%)			Average Computing Time (Second)		
Task	Goal-Biased RRT	CSA-RRT	CS-RRT	Goal-Biased RRT	CSA-RRT	CS-RRT
Task 1	100	100	100	0.015	0.019	0.015
Task 2	94	96	98	0.097	0.096	0.071
Task 3	94	94	96	0.143	0.087	0.083
Task 4	100	100	100	0.013	0.016	0.013
Average	97	97.5	98.5	0.067	0.0545	0.0455

## Data Availability

Not applicable.
